# Whole-genome sequencing identified novel mutations in a Chinese family with lynch syndrome

**DOI:** 10.3389/fonc.2023.1036356

**Published:** 2023-02-16

**Authors:** Wan He, Shaowei Dong, Jing Shen, Jiutong Wu, Pan Zhao, Dongbing Li, Dongliang Wang, Na Tang, Chang Zou

**Affiliations:** ^1^ Department of Oncology, Shenzhen People’s Hospital, The Second Clinical Medical College, Jinan University, Shenzhen, Guangdong, China; ^2^ Department of Oncology, Shenzhen People’s Hospital, The First Affiliated Hospital, Southern University of Science and Technology, Shenzhen, Guangdong, China; ^3^ Department of Hematology and Oncology, Shenzhen Children’s Hospital of China Medical University, Shenzhen, Guangdong, China; ^4^ Pathology Department, Shenzhen Baoan Women’s and Children’s Hospital, Shenzhen, Guangdong, China; ^5^ School of Medicine, The First Affiliated Hospital, Southern University of Science and Technology, Shenzhen, Guangdong, China; ^6^ Department of Medicine, ChosenMed Technology Beijing Co., Ltd, Beijing, China; ^7^ Department of Pathology, Shenzhen People’s Hospital, The Second Clinical Medical College, Jinan University, Shenzhen, Guangdong, China; ^8^ Department of Pathology, Shenzhen People’s Hospital, The First Affiliated Hospital, Southern University of Science and Technology, Shenzhen, Guangdong, China; ^9^ School of Medicine, Life and Health Sciences, The Chinese University of Hong Kong (Shenzhen), Shenzhen, Guangdong, China

**Keywords:** Lynch syndrome, whole genome sequencing, MMR pathway, MSH2, FSHR

## Abstract

**Background:**

Lynch syndrome (LS) is caused by a germline mutation in one of the mismatch repair genes (MLH1, MSH2, MSH6, and PMS2) or in the EPCAM gene. The definition of Lynch syndrome is based on clinical, pathological, and genetic findings. Therefore, the identification of susceptibility genes is essential for accurate risk assessment and tailored screening programs in LS monitoring.

**Patients and methods:**

In this study, LS was diagnosed clinically in a Chinese family using Amsterdam II criteria. To further explore the molecular characteristics of this LS family, we performed whole genome sequencing (WGS) to 16 members in this family and summarized the unique mutational profiles within this family. We also used Sanger sequencing technology and immunohistochemistry (IHC) to verify some of the mutations identified in the WGS analysis.

**Results:**

We showed that mutations in mismatch repair (MMR) related genes, as well as pathways including DNA replication, base excision repair, nucleotide excision repair, and homologous recombination were enhanced in this family. Two specific variants, MSH2 (p.S860X) and FSHR (p.I265V) were identified in all five members with LS phenotypes in this family. The MSH2 (p.S860X) variant is the first reported variant in a Chinese LS family. This mutation would result in a truncated protein. Theoretically, these patients might benefit from PD-1 (Programmed death 1) immune checkpoint blockade therapy. The patients who received nivolumab in combination with docetaxel treatments are currently in good health.

**Conclusion:**

Our findings extend the mutation spectrum of genes associated with LS in MLH2 and FSHR, which is essential for future screening and genetic diagnosis of LS.

## Introduction

Lynch syndrome (LS) is an autosomal dominant syndrome linked to a variety of cancers of the colon, endometrium, ovary, and others (1, 2). LS is mainly caused by germline and epistatic mutations in the human mismatch repair (MMR) gene (3). Maintaining genomic stability is a key function of the MMR protein (4). During DNA replication, repair, and recombination, the MMR system monitors and corrects errors (5). Several factors contribute to MMR, including MutS homolog 2 (MSH2), MutL homolog 1 (MLH1), MutS homolog 6 (MSH6), post-meiotic segregation increased 2 (PMS2), and epithelial cell adhesion molecule (EPCAM). Clinical, pathological, and genetic findings are used to diagnose LS (6). For clinical monitoring of carriers and genetic testing of relatives at high risk, it is therefore important to detect LS-related mutations (7).

LS is typically diagnosed clinically based on Amsterdam or Bethesda criteria (3). Patients with LS are typically screened for mutations in the MMR pathway using genetic testing (8). MLH1 and MSH2 mutations are most prevalent in LS (90%), MSH6 (10%), and PMS2 mutations are less frequency (9, 10).

This study aims to elucidate which variants of the MMR gene could provide a more accurate risk assessment or predictive test for existing ‘healthy’ members of affected families. Our study investigated LS due to mutations in a family using whole genome sequencing (WGS) and Sanger sequencing.

## Materials and methods

### Patient and ethical statements

In Shenzhen People’s Hospital’s Department of Medical Oncology, a four-generation Chinese family was diagnosed and treated for LS. According to the Amsterdam II criteria, clinical testing reports, and detailed family history, oncologists made the clinical diagnosis of LS. Informed consent was obtained from all four generations of Chinese family members participating in this study. In accordance with the Declaration of Helsinki, the Ethics Committee of Shenzhen People’s Hospital reviewed and approved our study.

### DNA extraction

A QIAamp DNA Blood Midi Kit (Qiagen; Valencia, CA, USA) was used to extract DNA from participants’ peripheral blood for whole-genome sequencing and Sanger sequencing.

### Whole genome sequencing

Covaris-focused ultrasound (Covaris, MA, USA) was used to shear DNA and 6 cycles of PCR were used to enrich for fragments of DNA. Agilent 2100 Bioanalyzer was used to analyze the size distribution of the library. A 150bp paired-end read was generated from raw DNA libraries using Illumina Hiseq.

### Data processing

Trimmomatic (version 0.36) was used to discard raw reads contaminated with adapters and low-quality/unidentified nucleotides. Quality-controlled reads were compared to the UCSC (University of California, Santa Cruz) human reference genome (GRCh37) using BWA software (11). PCR duplicates were removed and bam files were indexed using Samtools and Picard. In order to generate the final BAM (the binary version of a SAM file) file, GATK (The Genome Analysis Toolkit) was used to recalibrate the base quality (12). GATK was used to identify single nucleotide variants (SNVs) in the germline, and ANNOVAR (ANNOtate VARiation) was used to annotate and prioritize those variants (13). SIFT, PolyPhen2, and MutationTaster were used to assess the pathogenicity of missense variants. All variants identified in this study were manually checked using Integrative Genomics Viewer (IGV version 2.3.86) and only variants in the coding and splice regions were considered (14). Copy number variants (CNVs) and structural variants were detected using Control-FREEC and Breakdancer, respectively (15, 16).

### Sanger sequencing

Sanger sequencing was used to validate the candidate variants identified above.

### Immunohistochemistry

The expression of proteins was detected using immunohistochemistry (17). The DAKO EnVision system was used for immunoperoxidase staining. MLH1 (Roche, Shanghai, China), MSH2 (Roche, Shanghai, China), MSH6 (Roche, Shanghai, China), and PMS2 (Roche, Shanghai, China) were used as primary antibodies. Immunoglobulin G (IgG, Heavy Chain + Light chain) Mouse universal immunohistochemistry antibody (Roche, Shanghai, China) was used as a secondary antibody.

### Gene ontology biological process enrichment analysis

Gene Ontology (GO) enrichment analysis was performed using R “clusterProfiler” package using genes with >=20 mutations as input. A p.adj of 0.05 was used as cutoff for statistical significance.

### Statistical analysis

Statistical analysis was performed with R (version 3.6.3) (18–20). Results expressed as mean ± SD (Standard Deviations) were analyzed using the Student’s t.test. Differences were considered significant when P < 0.05.

## Results

### An LS pedigree of four generations

**Figure 1:**
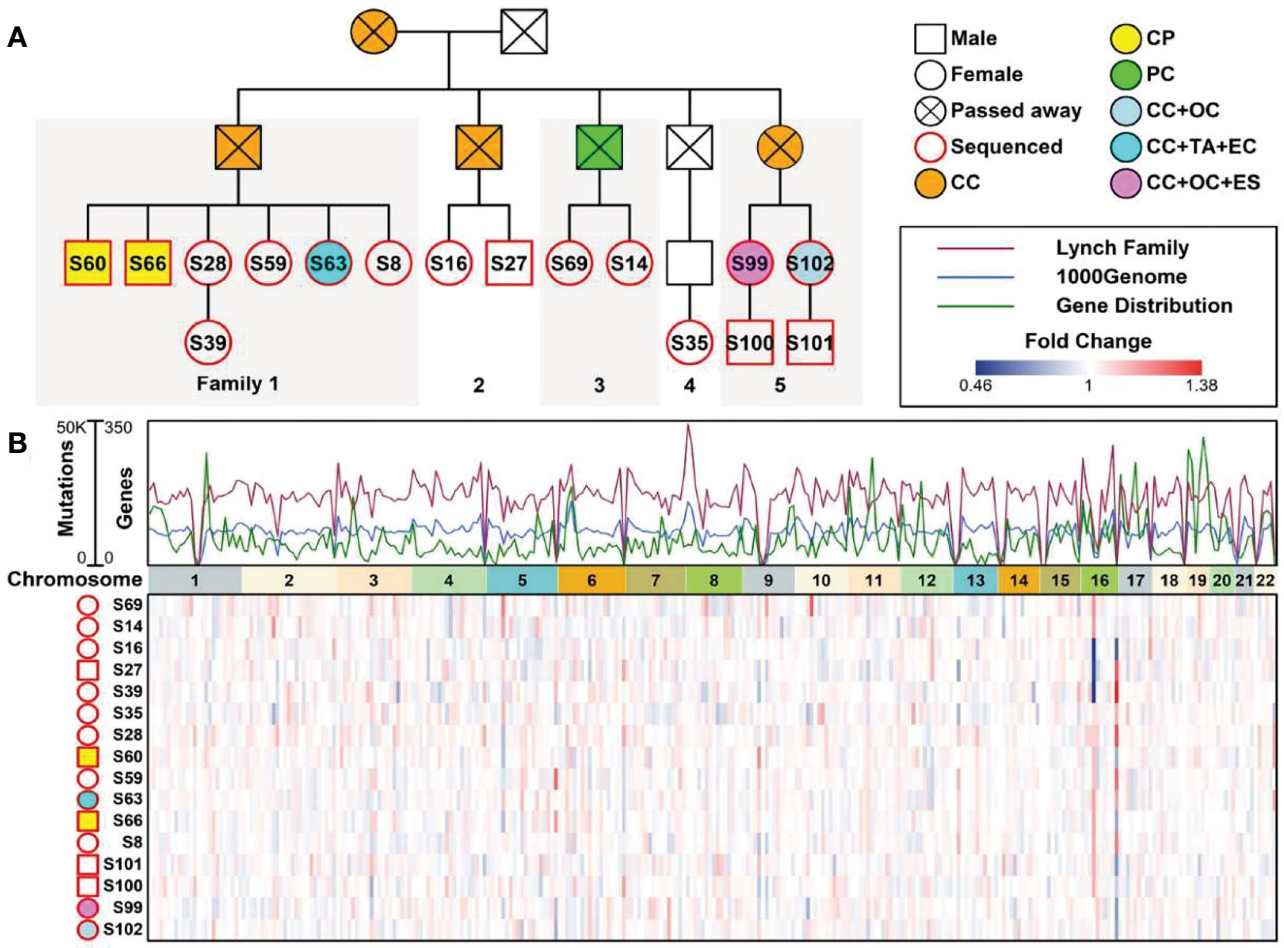
A Chinese family tree with LS. **(A)** An example of a Chinese pedigree with LS. Squares indicate males, circles indicate females. Squares and circles indicate males and females, respectively. Solid symbols indicate LS members, hollow symbols indicate unaffected individuals. S99 is the proband. CC, Colorectal Cancer; CP, Colorectal Polyps; PC, Pancreatic Cancer; OC, Ovarian Cancer; TA, Tubular Adenocarcinoma; EC, Endometrial Cancer; ES, Endometrial Sarcoma. **(B)** Combined plot representing the mutation status of 16 sequenced samples across 22 chromosomes.

A 53-year-old female with a personal and family history matching Lynch syndrome phenotype was involved in this study as the proband (S99 in **Figure 1A**). At the age of 49, this patient was diagnosed with endometrial sarcoma (**Supplementary Figures 1A–C**), ovarian cancer (**Supplementary Figures 1D–F**), and colorectal cancer (**Supplementary Figure 1G, H**). We further investigated the proband’s 24 relatives within four generations, as illustrated in **Figure 1A**. Among these 24 relatives, 1 female ancestor in generation 1 was diagnosed with colorectal cancer; 4 out of 5 ancestors from generation 2 were diagnosed with colorectal cancer or pancreatic cancer; 5 out of 13 participants in generation 3 were diagnosed with one or more than one types of the following diseases: patient 1 (S60) with colorectal polyps; patient 2 (S66) with colorectal polyps; patient 6 (S63) with colorectal cancer, tubular adenocarcinoma, and endometrial cancer; patient 13 (S99) with colorectal cancer, ovarian cancer, and endometrial sarcoma; patient 15 (S102) with colorectal cancer and ovarian cancer. We further performed whole genome sequencing to the peripheral blood samples of 16 members from this family (as shown in **Figure 1B** and listed in **Table 1**) and their SNP (single nucleotide polymorphism) and InDel (Insertion and deletion) distribution profiles were shown in **Supplementary Figure 2**.

**Table 1:** The clinical characteristics of LS patients.

Name	Subjects	Sex	Status	Age	Age of Onset	Tumor Type	Treatment	MSI	IHC	Lynch Syndrome
Patient 1	60	male	alive	51	47	CP	NA	NA	NA	Yes
Patient 2	66	male	alive	56	52	CP	NA	NA	NA	Yes
Patient 3	28	male	alive	66	NA	NA	NA	NA	NA	No
Patient 4	39	female	alive	48	NA	NA	NA	NA	NA	No
Patient 5	59	female	alive	58	NA	NA	NA	NA	NA	No
Patient 6	63	female	alive	58	50	CC, TA, EC	S	MSI-H		Yes
Patient 7	8	female	alive	62	NA	NA	NA	NA	NA	No
Patient 8	16	female	alive	61	NA	NA	NA	NA	NA	No
Patient 9	27	male	alive	55	NA	NA	NA	NA	NA	No
Patient 10	69	female	alive	58	NA	NA	NA	NA	NA	No
Patient 11	14	female	alive	53	NA	NA	NA	NA	NA	No
Patient 12	35	female	alive	21	NA	NA	NA	NA	NA	No
Patient 13	99	female	alive	53	49	CC, OC, ES	S+CH+IO	MSI-H	pMMR	Yes
Patient 14	100	male	alive	33	NA	NA	NA	NA	NA	No
Patient 15	102	female	alive	50	33	CC, OC	S+IO	MSI-H	dMMR	Yes
Patient 16	101	male	alive	28	NA	NA	NA	NA	NA	No

CC, Colorectal Cancer; CP, Colorectal Polyps; OC, Ovarian Cancer; TA, Tubular Adenocarcinoma; EC, Endometrial Cancer; ES, Endometrial Sarcoma; IHC- 4, Immunohistochemistry (MLH1, MSH2, MSH6, and PMS2); S, surgery; CH, chemotherapy; IO, immunotherapy; MSI-H, Microsatellite instability-high; pMMR, MMR proficient; dMMR, MMR deficient.

### The mutational profiles of members from this LS family

**Figure 2:**
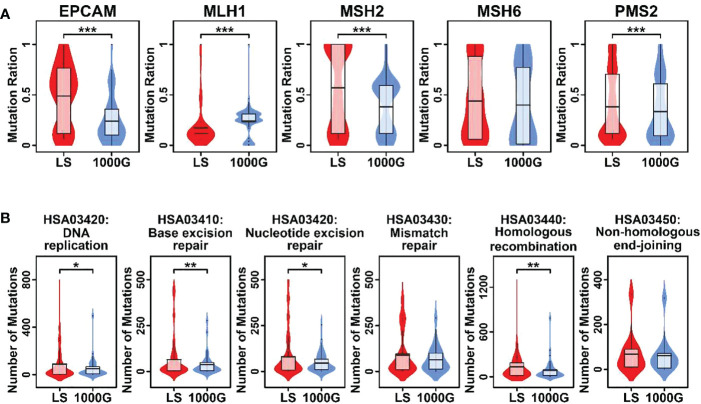
Violin plots representing mutational burdens in LS-related genes and MMR pathways. **(A)** Comparison of mutation numbers in 5 genes in this LS family and 1000genomes. **(B)** Comparison of mutation numbers in genes relating to 6 pathways in this LS family and 1000genomes. A student t.test was performed for each comparison. (*p < 0.05; **p < 0.01; ***p < 0.001).

To compare the mutational profiles of LS family members with normal profiles, in this study, we involved mutational profiles from 1000geneomes (www.internationalgenome.org) as background control. The mutation frequencies of EPCAM, MSH2, and PMS2 genes were significantly higher in LS family members compared to those in 1000genome profiles, and the mutation frequency of MLH1 gene was significantly lower in LS family members compared to it in 1000genome profiles (**Figure 2A**). Regarding MMR related pathways, pathways involved in DNA replication, base excision repairs, nucleotide excision repair, and homologous recombination had significantly higher numbers of mutations in LS family members compared to these in 1000genome profiles, as shown in **Figure 2B**.

**Figure 3:**
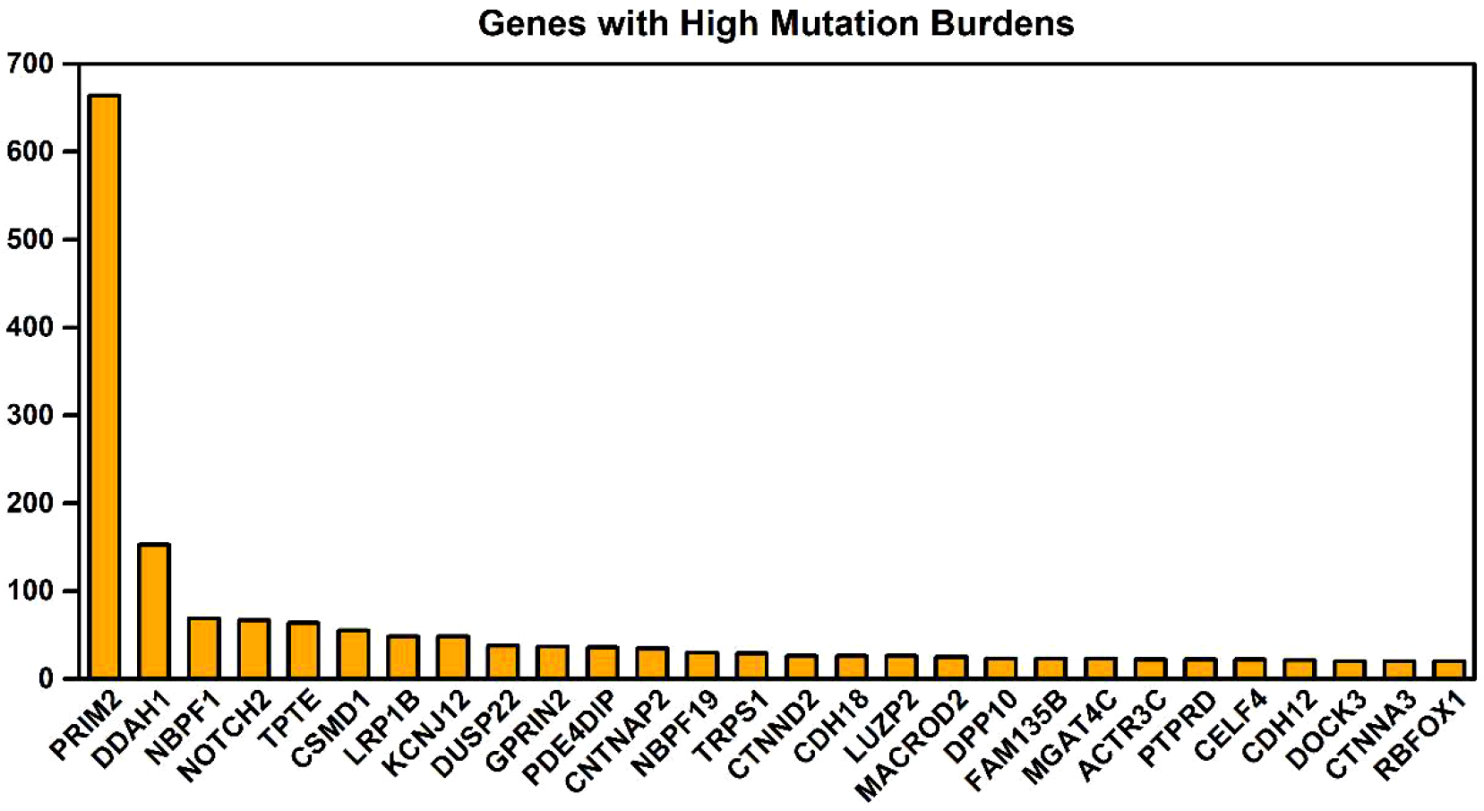
Genes with high mutational burdens in this LS family members.

The key gene mutations across the family were listed in **Table 2**. The top 28 genes with high mutation burdens (over 20) were listed in **Figure 3**. The top enriched GO BP (biological process) terms of these genes were listed in **Table 3**.

**Table 2:** Key gene mutation across the family.

Chromosome	Position	RS Number	Reference	Alteration	Gene	Type
1	17087541	rs113982165	GGTGCT	G	MST1L	Frameshift_deletion
4	140811063	rs774201781	TTGCTGCTGCTGC	T	MAML3	Frameshift_deletion
4	155244401	rs140019361	TTTTG	T	DCHS2	Frameshift_deletion
13	78272267	rs201380414	T	TGG	SLAIN1	Frameshift_insertion
7	151945071	rs150073007	G	GT	KMT2C	Stopgain
1	144915561	rs1778111	G	A	PDE4DIP	Stopgain
1	145075683	rs2762779	C	T	PDE4DIP	Stopgain
21	10942756	rs1810540	G	A	TPTE	Stopgain

**Table 3:** Enriched analysis of genes with high mutation burdens.

Enriched analysis	P value
Regulation of non-membrane spanning protein tyrosine kinase activity	0.01
Cell-cell adhesion	0.027
Cell-cell junction assembly	0.027
Cell junction assembly	0.027
Cell junction organization	0.027
Adherens junction organization	0.027
Peptidyl-tyrosine dephosphorylation	0.027
Cell-cell junction organization	0.033
Calcium-dependent cell-cell adhesion *via* plasma membrane cell adhesion molecules	0.041
Cell-cell adhesion mediated by cadherin	0.041
Cell adhesion	0.043
Biological adhesion	0.043
Multicellular organismal signaling	0.043

### Unique mutational features of 5 members with LS phenotype

**Figure 4:**
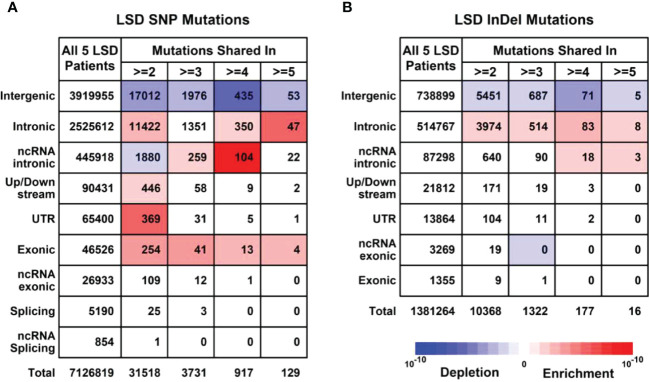
Mutational profiles in LSD members (Family members with LS phenotype, n = 5). **(A)** Details of SNP (Single nucleotide polymorphisms) profiles in 5 LSD members. **(B)** Details of InDel (Insertion and Deletion mutations) profiles in 5 LSD members. Enrichment/depletion estimation was performed by hypergeometric distribution using data from LSN members as background.

To further explore the unique mutational profiles of members with LS phenotype, we divided these 16 members into two groups, 5 members with LS phenotype (LSD including S60: CP; S66: CP; S63:CC+TA+EC; S99:CC+OC+ES; S102:CC+OC) and 11 members without LS phenotype (LSN including S28, S100, S35, S14, S69, S27, S16, S8, S63, S59, S39, S28), as illustrated in **Supplementary Figure 3**. Regarding SNP mutations, the number of different types of mutations shared in the different numbers of LSD members was listed in **Figure 4A**. Specifically, exonic SNP mutations were enriched in LSD members compared to these in LSN members (red); regarding InDel mutations, the number of different types of mutations shared in the different numbers of LSD members was listed in **Figure 4B**. Specifically, intronic mutations were enriched in LSD members compared to these in LSN members (red).

**Figure 5:**
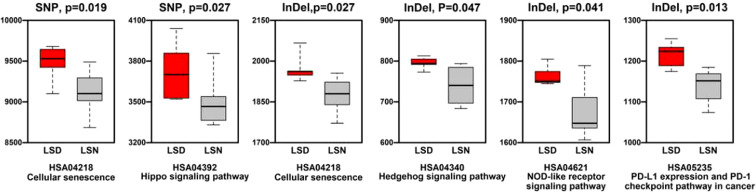
Comparison of mutation numbers in genes relating to KEGG pathways between LSD members and LSN members.

Generally, the level of CNVs (Copy Number Variations) in LSD members was significantly higher than this in LSN members (*p* < 0.05), as shown in **Supplementary Figure 4**. Regarding KEGG (Kyoto Encyclopedia of Genes and Genomes) enrichment analysis, pathways relating to cellular senescence, hippo signaling pathway, NOD (Nucleotide oligomerization domain)-like receptor signaling pathway, and PD-L1 (Programmed death ligand-1) expression/PD-1(Programmed death-1) checkpoint pathway in cancer had more SNP/InDel mutations in LSD members compared to these in LSN members (**Figure 5**).

**Figure 6:**
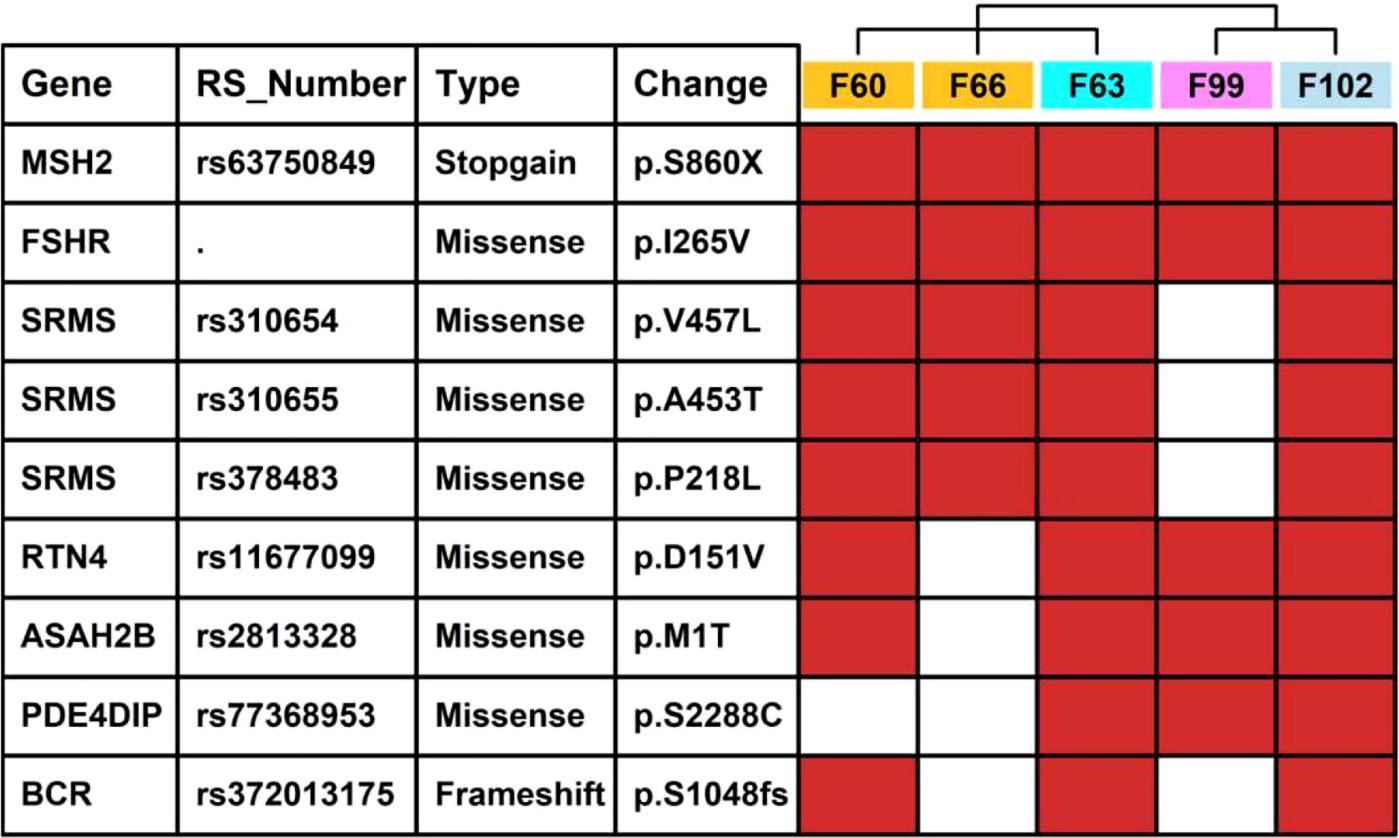
Key mutations in 5 LSD patients. The red rectangle represents that the specific mutation is present in the individual sample.

We also summarized missense/stopgain/frameshift mutations that were shared in more than 3 LSD members, as illustrated in **Figure 6**. There were 2 mutations (MSH2.p.860X, FSHR.p.I265V) shared in all 5 LSD members, 5 mutations (SRMS.p.V457L, SRMS.p.A453T, SRMS.p.P218L, RTN4.p.D151V and ASAH2B.p.S2288C) that were shared in 4 LSD members, 2 mutations (PDE4DIP.p.S2288C, BCR.p.S1048fs) that were shared in 3 LSD members.

### The validation of the mutations

**Figure 7:**
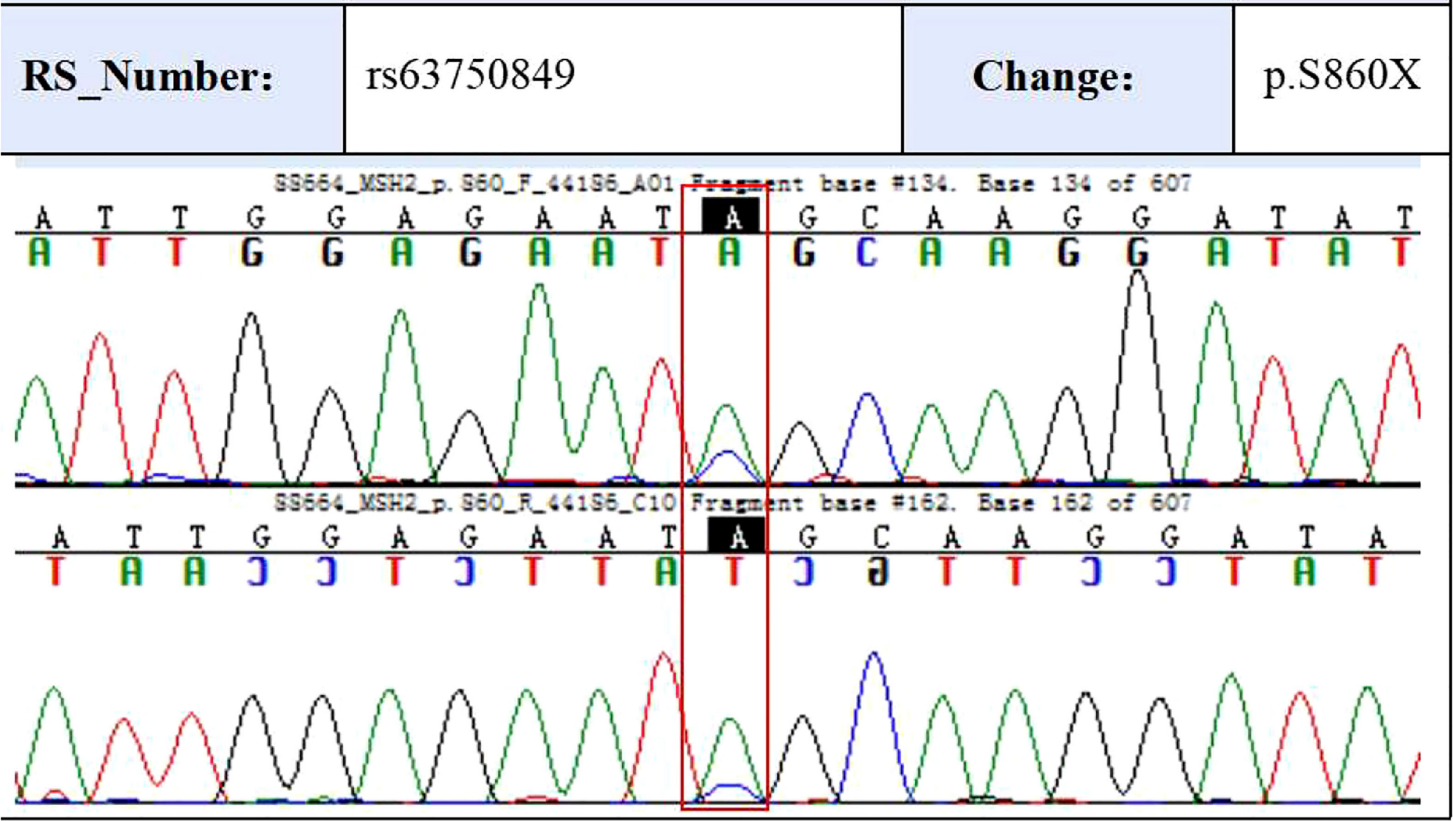
Sanger sequencing confirms mutation in MSH2 (p.S860X) in proband patient.

**Figure 8:**
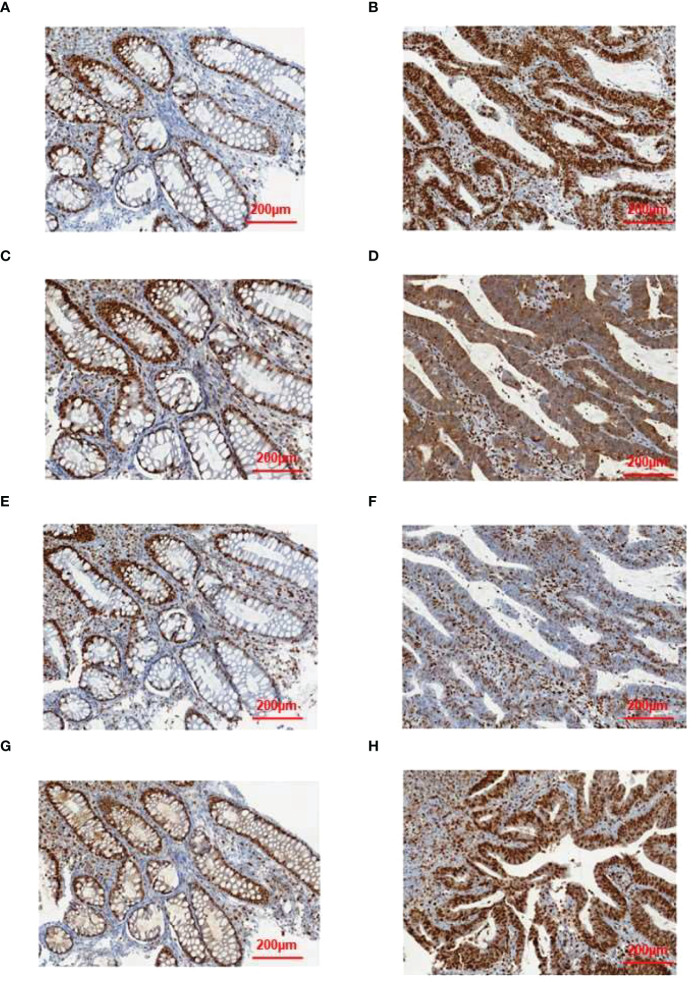
Results of immunohistochemical validation. The expression of **(A)** MLH1, **(C)** MSH2, **(E)** MSH6, and **(G)** PMS2 in normal tissue, and the expression of **(B)** MLH1, **(D)** MSH2, **(F)** MSH6, and **(H)** PMS2 in tumor tissue.

To validate some of these variants, we performed sanger sequencing to samples of patient 13 (S99, the proband), and the result was shown in **Figure 7**. MSH2 (p.S860X) mutation was verified in tumor samples from patient 13. We also found higher expression of MMR related proteins including MLH1, MSH2, MSH6, and PMS2 in the tumor tissues of patient 13 compared to these in the paraCancer tissues, as shown in **Figure 8**.

### Therapeutic actions involved in the treatment of LSD members in this family

The proband (patient 13, S99) was diagnosed with ovarian cancer and endometrial sarcoma at age 49 and had radical surgery afterward. The colorectal cancer was accidentally found when scanned at baseline radiological evaluation before delivering adjuvant treatment. According to the Amsterdam II criteria, we examined her gene mutation profiles and found the MSH2 mutation in her peripheral blood samples. After that, this patient received six cycles of nivolumab in combination with docetaxel and cisplatin plus fluorouracil followed by 8 cycles of single-agent nivolumab. The patient is currently in good condition.

Two female patients with colorectal cancer as well as germline MSH2 mutations received endoscopic submucosal dissection. Among them, one female patient was subsequentially diagnosed with colorectal cancer, tubular adenocarcinoma, and endometrial cancer (patient 6, S63), and these tumors were all resected radically; another female patient, who is the sister of the proband, was diagnosed with colorectal cancer (resected in 2003) and ovarian cancer (resected in 2018). There was a recurrence of colorectal cancer in patient 6 in early 2022, during which this patient received 8 cycles of pembrolizumab as chemotherapy treatment.

## Discussion

The Lynch syndrome (LS) is an autosomal dominant disorder linked to a high risk of cancer, especially colorectal cancer (21). LS is difficult to diagnose due to the following reasons: the diagnosis of LS is mainly based on clinical criteria; currently, it is hard to obtain family information; large-scope clinical phenotype information such as polyposis information is not available (3). As a result, the rate of LS diagnosis is far behind the actual incidence (9). To achieve a better therapeutic effect, as well as a good prognosis, early diagnosis is essential. Large-scale screening programs might be more beneficial for those who carry the causative mutations, while not so necessary for those who do not carry them. The identification of mutations that cause LS in LS families is useful for genetic counseling and disease management.

MSH2 was first mapped to 2p21 in 1993, and several deleterious mutations within this gene were identified in LS families (22). Subsequently, many mutations relating to MMR genes (MLH1, MSH2, MSH6, and PMS2) were also identified in the LS family. MSH2 variant c.2635-2A>G is pathogenic because it leads to an alteration of the typical splice site, resulting in an abnormal form of the protein product (MSH2) (23). Zajo et al. reported a study of childhood LS-associated colorectal carcinomatosis caused by a pathogenic germline mutation in MSH2 (c.1786_1788delAAT (p.Asn596del)) (24). Cariola et al. reported a rare variant MSH2 c(c.2635-2A>G) which would affect the splice site consensus sequence of intron 15 in MSH2, and concluded the potential pathogenic role of this variant (25). Follicle-stimulating hormone receptor (FSHR), expressed in vascular endothelial cells of different malignancies, has recently been investigated as a potential pan-receptor for cancer therapy (26–28). A missense mutation (p.I265V) leading to an amino acid switch from isoleucine (amino acid with hydrophobic side chain) to similar valine (amino acid with hydrophobic side chain) might not cause a dramatic structural change in FSHR protein. So far, the relationship between FSHR and LS has not been reported, and it might need further effort in exploring their connections.

In this study, we performed WGS on 16 members of this LS family. First, we tried to answer why members of this family tended to have LS. We found that mutational levels relating to MMR pathways were enhanced, and mutational levels in pathways such as DNA DNA replication, base excision repair, nucleotide excision repair and homologous recombination were also enhanced. Second, we tried to answer why these 5 LSD members had LS phenotype instead of the other 11 LSN members. Two mutations (MSH2.p.S860X and FSHR.p.I265V) were shared among all these 5 LSD members other than the 11 LSN members. Based on the HNPCC mutation database, the germline mutation MSH2 (p.S860X) was reported to be found in the investigated HNPCC patients (29), which was the first report of a germline variant of MSH2 (p.S860X) in a Chinese population. Sanger sequencing confirmed that this predisposed individual carried MSH2 (p.S860X).

We hypothesize that the MSH2 mutation (p.S860X) is the primary cause of Lynch syndrome in this family and plays a significant role in its onset. We speculate that the other high-frequency mutations found may not play a major role in the development of LS. Further validation is needed for the role played by some variants in tumor-associated genes in members of this family.

## Conclusion

In conclusion, our study provides a preliminary exploration of LS pathogenesis from the perspective of a complete LS family pedigree. Our results suggest key mutations including MSH2 (p.S860X) and FSHR (p.I265V), as well as increased mutations in MMR-related pathways could also contribute to the incidence of LS. The data presented in the study are deposited in GSA Human database (https://ngdc.cncb.ac.cn/gsa-human/), accession number HRA003905.

## Data availability statement

The data presented in the study are deposited in GSA Human database (https://ngdc.cncb.ac.cn/gsa-human/), accession number HRA003905.

## Ethics statement

The studies involving human participants were reviewed and approved by the ethics committee of Shenzhen People’s Hospital. The patients/participants provided their written informed consent to participate in this study.

## Author contributions

WH and CZ conceived the research idea. WH and NT prepared and wrote the manuscript. SD, DL and DW performed data analysis. WH, JS, JW and PZ collected the clinical samples. JW, NT and CZ revised the manuscript. All authors contributed to the article have approved the submitted version.

## References

[1] Aarnio M Sankila R Pukkala E Salovaara R Aaltonen LA de la Chapelle A. Cancer risk in mutation carriers of DNA-mismatch-repair genes. Int J Cancer (1999) 81:214–8. doi: 10.1002/(sici)1097-0215(19990412)81:2<214::aid-ijc8>3.0.co;2-l 10188721

[2] Shao W-H Wang C-Y Wang L-Y Xiao F Xiao D-S Yang H. A hereditable mutation of MSH2 gene associated with lynch syndrome in a five generation Chinese family. Cancer Manag Res (2020) 12:1469–82. doi: 10.2147/CMAR.S222572 PMC7051253 32161499

[3] Liu Y Wang M Chen Q Zheng Q Li G Cheng Q. A novel heterozygous large deletion of MSH6 gene in a Chinese family with lynch syndrome. Gene (2019) 704:103–12. doi: 10.1016/j.gene.2019.04.011 30974197

[4] Sameer AS Nissar S Fatima K. Mismatch repair pathway: Molecules, functions, and role in colorectal carcinogenesis. Eur J Cancer Prev Off J Eur Cancer Prev Organ (2014) 23:246–57. doi: 10.1097/CEJ.0000000000000019 24614649

[5] Casey G Lindor NM Papadopoulos N Thibodeau SN Moskow J Steelman S. Conversion analysis for mutation detection in MLH1 and MSH2 in patients with colorectal cancer. JAMA (2005) 293:799–809. doi: 10.1001/jama.293.7.799 15713769 PMC2933041

[6] Ghaedi H Ramsheh SM Omidvar ME Labbaf A Alehabib E Akbari S. Whole-exome sequencing identified a novel mutation of MLH1 in an extended family with lynch syndrome. Genes Dis (2020) 7:614–9. doi: 10.1016/j.gendis.2019.07.011 PMC7729095 33335961

[7] Janavicius R Elsakov P. Novel germline MSH2 mutation in lynch syndrome patient surviving multiple cancers. Hered Cancer Clin Pract (2012) 10:1. doi: 10.1186/1897-4287-10-1 22234272 PMC3275504

[8] Lynch HT Snyder CL Shaw TG Heinen CD Hitchins MP. Milestones of lynch syndrome: 1895-2015. Nat Rev Cancer (2015) 15:181–94. doi: 10.1038/nrc3878 25673086

[9] Giardiello FM Allen JI Axilbund JE Boland CR Burke CA Burt RW. Guidelines on genetic evaluation and management of lynch syndrome: A consensus statement by the US multi-society task force on colorectal cancer. Gastroenterology (2014) 147:502–26. doi: 10.1053/j.gastro.2014.04.001 25043945

[10] Rossi BM Palmero EI López-Kostner F Sarroca C Vaccaro CA Spirandelli F. A survey of the clinicopathological and molecular characteristics of patients with suspected lynch syndrome in Latin America. BMC Cancer (2017) 17:623. doi: 10.1186/s12885-017-3599-4 28874130 PMC5586063

[11] Li H Durbin R. Fast and accurate short read alignment with burrows-wheeler transform. Bioinformatics (2009) 25:1754–60. doi: 10.1093/bioinformatics/btp324 PMC2705234 19451168

[12] DePristo MA Banks E Poplin R Garimella KV Maguire JR Hartl C. A framework for variation discovery and genotyping using next-generation DNA sequencing data. Nat Genet (2011) 43:491–8. doi: 10.1038/ng.806 PMC3083463 21478889

[13] Wang K Li M Hakonarson H. ANNOVAR: Functional annotation of genetic variants from high-throughput sequencing data. Nucleic Acids Res (2010) 38:e164. doi: 10.1093/nar/gkq603 20601685 PMC2938201

[14] Robinson JT Thorvaldsdóttir H Winckler W Guttman M Lander ES Getz G. Integrative genomics viewer. Nat Biotechnol (2011) 29:24–6. doi: 10.1038/nbt.1754 PMC3346182 21221095

[15] Boeva V Zinovyev A Bleakley K Vert J-P Janoueix-Lerosey I Delattre O. Control-free calling of copy number alterations in deep-sequencing data using GC-content normalization. Bioinformatics (2011) 27:268–9. doi: 10.1093/bioinformatics/btq635 PMC3018818 21081509

[16] Chen K Wallis JW McLellan MD Larson DE Kalicki JM Pohl CS. BreakDancer: An algorithm for high-resolution mapping of genomic structural variation. Nat Methods (2009) 6:677–81. doi: 10.1038/nmeth.1363 PMC3661775 19668202

[17] Yang Y Gu X Li Z Zheng C Wang Z Zhou M. Whole-exome sequencing of rectal cancer identifies locally recurrent mutations in the wnt pathway. Aging (Albany NY) (2021) 13:23262–83. doi: 10.18632/aging.203618 PMC8544332 34642262

[18] Chen J Tang H Li T Jiang K Zhong H Wu Y. Comprehensive analysis of the expression, prognosis, and biological significance of OVOLs in breast cancer. Int J Gen Med (2021) 14:3951–60. doi: 10.2147/IJGM.S326402 PMC8323863 34345183

[19] Lin Z Huang W Yi Y Li D Xie Z Li Z. LncRNA ADAMTS9-AS2 is a prognostic biomarker and correlated with immune infiltrates in lung adenocarcinoma. Int J Gen Med (2021) 14:8541–55. doi: 10.2147/IJGM.S340683 PMC8626860 34849000

[20] Yi W Shen H Sun D Xu Y Feng Y Li D. Low expression of long noncoding RNA SLC26A4 antisense RNA 1 is an independent prognostic biomarker and correlate of immune infiltrates in breast cancer. Med Sci Monit Int Med J Exp Clin Res (2021) 27:e934522. doi: 10.12659/MSM.934522 PMC8669971 34880202

[21] Hsieh P Zhang Y. The devil is in the details for DNA mismatch repair. Proc Natl Acad Sci U.S.A. (2017) 114:3552–4. doi: 10.1073/pnas.1702747114 PMC5389296 28356513

[22] Leach FS Nicolaides NC Papadopoulos N Liu B Jen J Parsons R. Mutations of a mutS homolog in hereditary nonpolyposis colorectal cancer. Cell (1993) 75:1215–25. doi: 10.1016/0092-8674(93)90330-s 8261515

[23] Pinheiro M Francisco I Pinto C Peixoto A Veiga I Filipe B. The nonsense mutation MSH2 c.2152C>T shows a founder effect in Portuguese lynch syndrome families. Genes Chromosomes Cancer (2019) 58:657–64. doi: 10.1002/gcc.22759 30968502

[24] Zajo K Colace SI Mouhlas D Erdman SH. Lynch syndrome-associated colorectal cancer in a 16-year-old girl due to a de novo MSH2 mutation. BMJ Case Rep (2020) 13(7):e233935. doi: 10.1136/bcr-2019-233935 PMC7332177 32611652

[25] Cariola F Disciglio V Valentini AM Lotesoriere C Fasano C Forte G. Characterization of a rare variant (c.2635-2A>G) of the MSH2 gene in a family with lynch syndrome. Int J Biol Markers (2018), 1724600818766496. doi: 10.1177/1724600818766496 29690800

[26] Olejar T Vetvicka D Boucek J Zabrodsky M Benes J Kabesova M. The FSHR expression in head and neck squamous cell cancer. a pilot immunohistochemical study. Anticancer Res (2020) 40:349–56. doi: 10.21873/anticanres.13959 31892586

[27] Papadimitriou K Kountourakis P Kottorou AE Antonacopoulou AG Rolfo C Peeters M. Follicle-stimulating hormone receptor (FSHR): A promising tool in oncology? Mol Diagn Ther (2016) 20:523–30. doi: 10.1007/s40291-016-0218-z 27392476

[28] Sun D Bai M Jiang Y Hu M Wu S Zheng W. Roles of follicle stimulating hormone and its receptor in human metabolic diseases and cancer. Am J Transl Res (2020) 12:3116–32. PMC7407683 32774689

[29] Krüger S Plaschke J Jeske B Görgens H Pistorius SR Bier A. Identification of six novel MSH2 and MLH1 germline mutations in HNPCC. Hum Mutat (2003) 21:445–6. doi: 10.1002/humu.9121 12655562

