# Supplementary material for: Whole-genome sequencing identified novel mutations in a Chinese family with lynch syndrome
Source: Front Oncol. 2023 Feb 16;13:1036356. doi: 10.3389/fonc.2023.1036356 (PMC9978139; doi:10.3389/fonc.2023.1036356)
Supplement: Supplementary file 1 [file DataSheet_1.pdf]

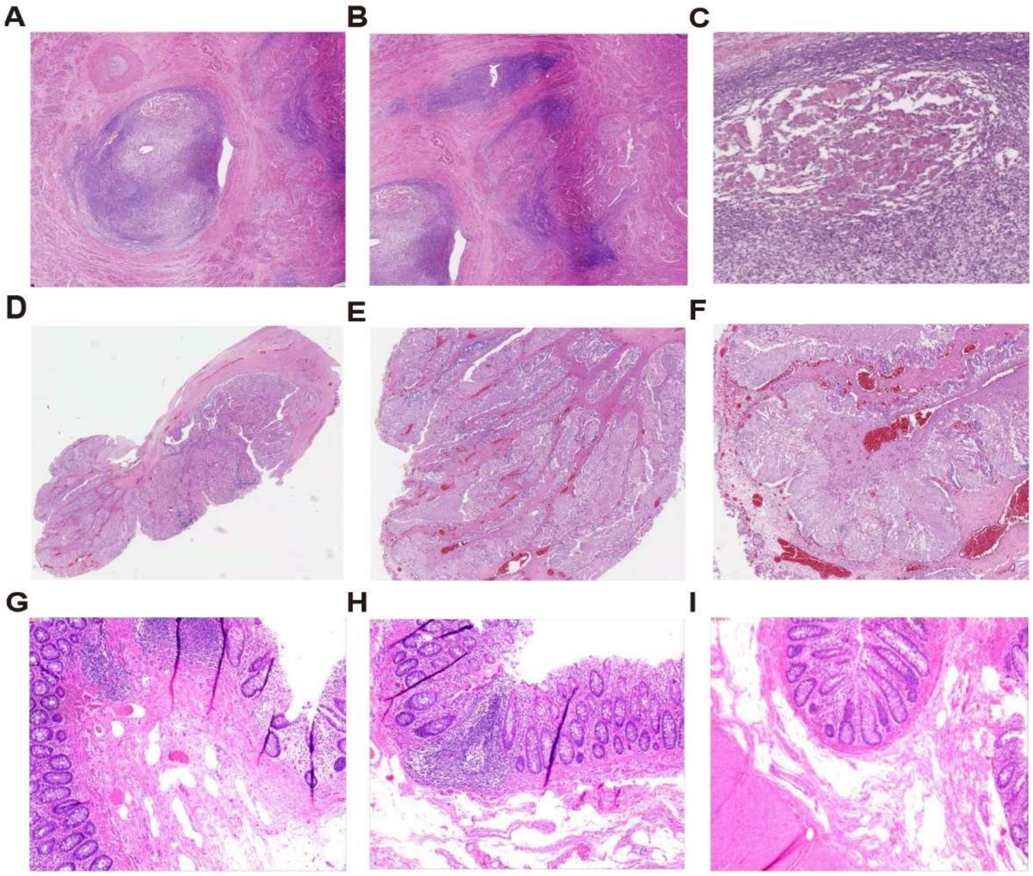

Supplementary Figure 1. Patient's pathology test results. (A), (B), and (C): pathological results of endometrial sarcoma; (D), (E), and (F): pathological results of ovarian cancer; (G), (H), and (I): pathological results of colorectal cancer.

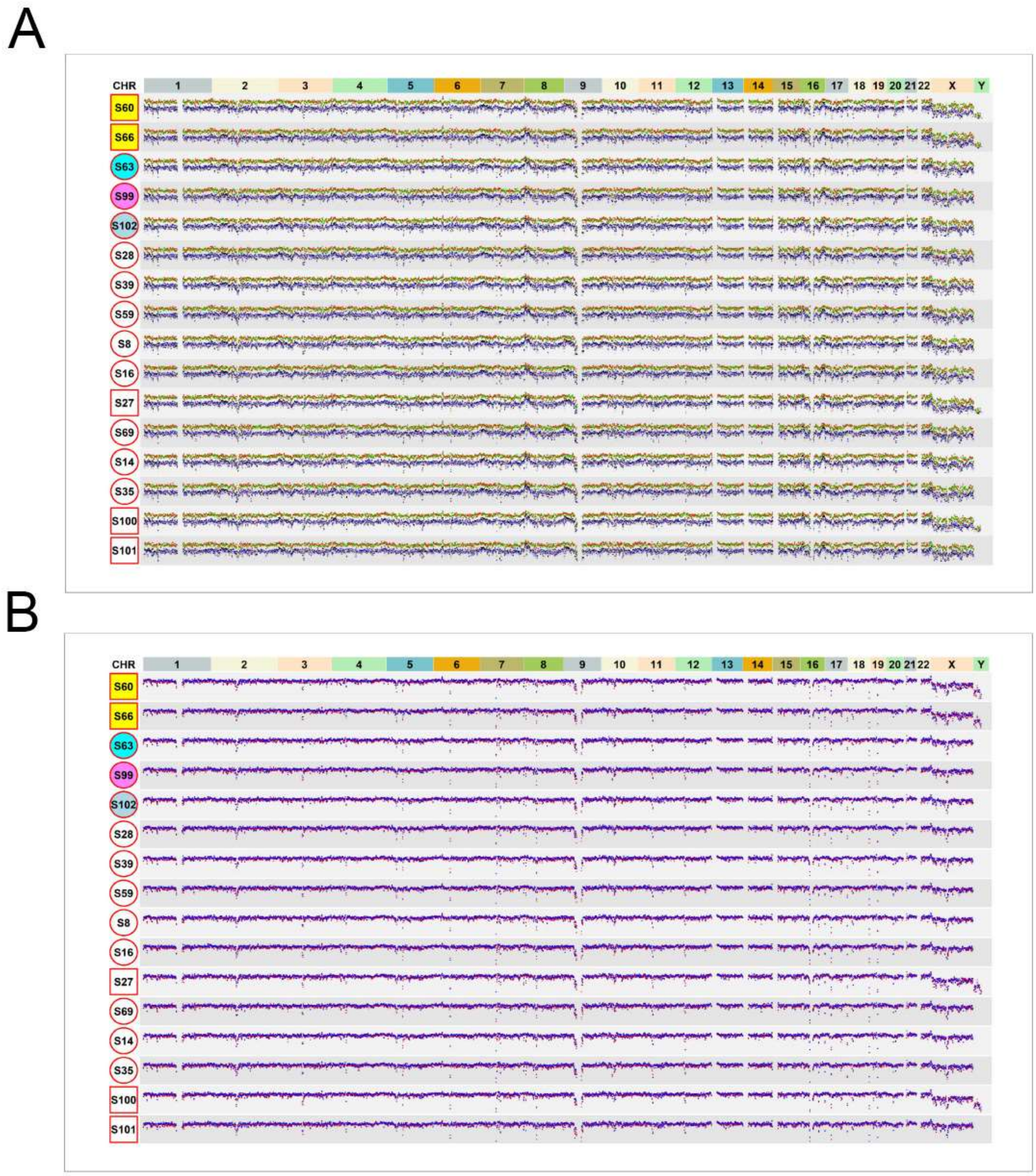

Supplementary Figure 2. Results of whole genome sequencing. (A) Rainfall plot illustrating the SNP distribution of all sequenced samples. (B) Rainfall plot illustrating the InDel distribution of all sequenced samples.

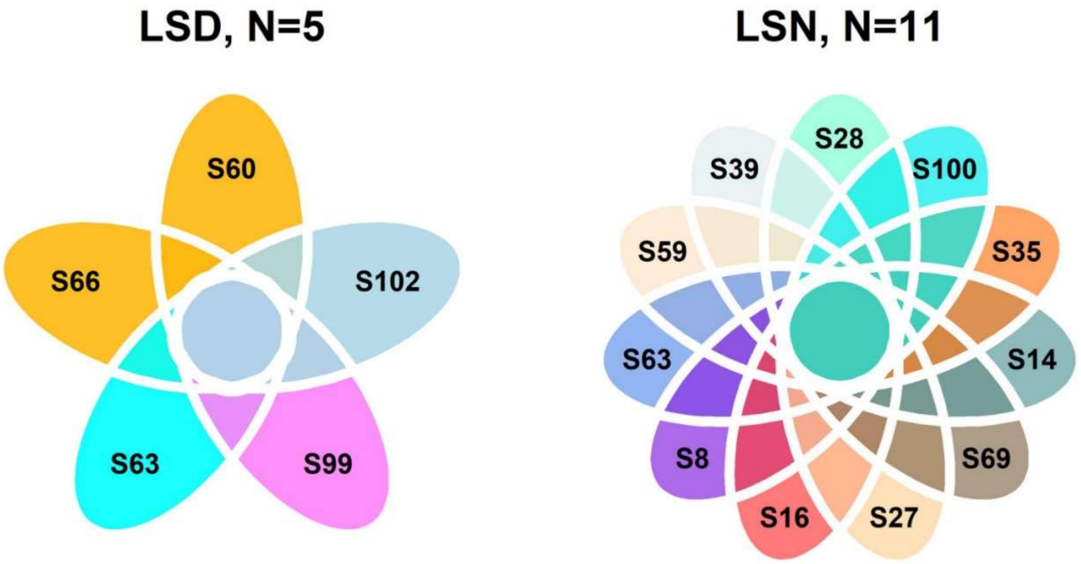

Supplementary Figure 3. Subgroups of patients and normal people in this family.

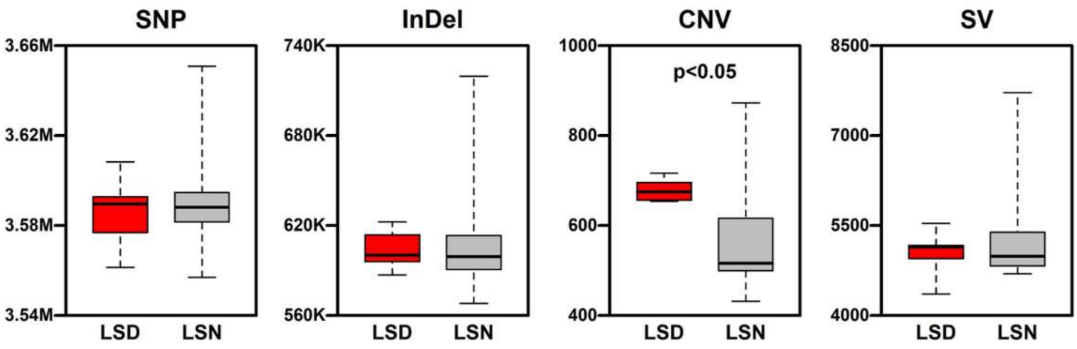

Supplementary Figure 4. Mutational profiles of LSD members (Family members with LS phenotype, n = 5) and LSN members (Family members without LS phenotype, n = 11). SNP: Single-nucleotide polymorphism; InDel: Insertion and deletion mutations; CNV: Copy number variation; SV: Structural variant. A student t.test was performed to each comparison.
